# Editorial: The mouth as a diagnostic window: unveiling the systemic implications of oral health

**DOI:** 10.3389/froh.2026.1910141

**Published:** 2026-07-07

**Authors:** Nicolas Marcelo Bertolini Novaes, Marinês Nobre-dos-Santos, Fabíola Galbiatti de Carvalho, Vanessa Pardi, Ramiro Mendonça Murata, Thaís Manzano Parisotto

**Affiliations:** 1 Laboratory of Clinical and Molecular Microbiology, University São Francisco, Bragança-Paulista, Brazil; 2 Department of Pediatric Dentistry, University of Campinas, Piracicaba, Brazil; 3 Department of Pediatric Dentistry, Federal University of Uberlândia, Uberlândia, Brazil; 4 Department of Foundational Science, ECU School of Dental Medicine, Greenville, NC, United States

**Keywords:** biomarkers, mouth, body fluids, health status, diagnosis

The oral cavity has traditionally been considered the primary site of oral diseases. However, growing evidence supports its role as a dynamic and integrative interface that reflects systemic health (Wu et al., Jaber et al., Liu et al., Cao et al., Nath et al., Katzenelson et al., Moldes et al.). Through complex interactions among immune responses, microbiome composition, and host-related factors, the oral cavity emerges as a non-invasive diagnostic window, capable of revealing not only early signs of systemic disturbances but also subtle physiological imbalances that may precede clinical manifestations (Wu et al., Jaber et al., Liu et al., Cao et al., Nath et al., Katzenelson et al., Moldes et al.).

This Research Topic comprises seven articles that collectively explore the oral cavity as a biological and diagnostic interface, encompassing topics such as inflammatory pathways, microbiome interactions, systemic disease associations, metabolic biomarkers, and clinical diagnostic challenges across diverse populations. Together, these studies provide a multidimensional perspective that links molecular mechanisms, clinical observations, and broader socio-environmental determinants of health, underscoring the diagnostic relevance of the oral cavity and its potential to inform our understanding of systemic health.

Inflammatory mechanisms are key links between oral and systemic health. In this context, Liu et al. demonstrated that orthodontic mechanical stress triggers a rapid and sustained rise in the levels of neutrophil extracellular traps (NETs) and pro-inflammatory cytokines, including IL-1β and IL-8, in saliva and gingival crevicular fluid. These results emphasize how responsive the oral immune microenvironment is to local stimuli and how effectively it reflects acute inflammatory changes. Expanding on this view, Moldes et al. found that periodontitis is significantly associated with leukoaraiosis, independent of major vascular risk factors, and is accompanied by elevated systemic levels of soluble tumor necrosis factor-like weak inducer of apoptosis (sTWEAK). This finding suggests a possible connection between chronic oral inflammation and neurological alterations. In addition, Wu et al. reported that tooth loss is associated with higher all-cause mortality in a large longitudinal cohort, with systemic inflammation, particularly high-sensitivity C-reactive protein (hs-CRP), partly mediating this relationship. Taken together, these findings show that oral inflammation is not merely a local condition but also a biologically relevant factor in systemic disease progression and long-term health outcomes.

In addition to inflammatory pathways, the oral microbiome emerges as a key mediator of systemic interactions. In a preliminary study, Katzenelson et al. demonstrated a significant correlation between oral and intestinal microbiota in children with acute appendicitis, with the Bacteroidetes phylum serving as a key taxonomic link. These findings reinforce the concept that bacterial shifts in one site may reflect or influence pathological processes along the oral–gut axis, particularly in the context of disease-associated microbial dysbiosis. Complementing this biological framework, Nath et al. explored the concept of the sociobiome, demonstrating that socio-economic disadvantage significantly shapes oral microbial diversity and composition. The authors’ findings indicate that a substantial proportion of the association between low income and dental caries is mediated by microbiome alterations, emphasizing that oral health outcomes are deeply embedded within social and environmental contexts. Together, these findings highlight the interplay between oral microbiome dynamics and socio-economic and environmental factors in shaping oral health outcomes and influencing disease susceptibility.

Metabolic regulation also represents an emerging and underexplored dimension of the oral-systemic connection. Jaber et al. highlighted in their review the potential role of calbindin-D28K, a calcium-binding protein involved in maintaining calcium homeostasis, in patients with chronic kidney disease. Alterations in calcium metabolism in these individuals were associated with changes in salivary composition and increased susceptibility to dental caries and periodontal disease. Although direct causal relationships remain to be fully elucidated, the authors suggest that calbindin-D28K may be a promising biomarker for monitoring oral-systemic interactions, thereby expanding the possibilities for non-invasive diagnostics.

From a clinical perspective, the oral cavity frequently serves as a sentinel site for systemic pathology, reinforcing its role as a valuable diagnostic interface in routine practice. Notably, Cao et al. described a case of delayed diagnosis of hypophosphatasia in which dental abnormalities, including early tooth loss, preceded the systemic diagnosis by over a decade. This highlights a critical gap and underscores the importance of dentists in identifying early signs of rare systemic conditions.

Collectively, the studies included in this Research Topic position the oral cavity as a central component of systemic health, integrating inflammatory, microbiological, metabolic, and socio-environmental dimensions. Rather than functioning as an isolated anatomical site, the oral cavity should be understood as a responsive and informative system capable of both reflecting and influencing systemic processes. This perspective supports the development of non-invasive diagnostic tools—such as salivary and microbiome biomarkers— while integrating Dentistry into interdisciplinary healthcare. Future research should focus on translating these insights into clinical protocols to enhance early disease detection, refine risk stratification, and ultimately lead to better patient outcomes.

